# Polyethersulfone Polymer for Biomedical Applications and Biotechnology

**DOI:** 10.3390/ijms25084233

**Published:** 2024-04-11

**Authors:** Monika Wasyłeczko, Cezary Wojciechowski, Andrzej Chwojnowski

**Affiliations:** Nalecz Institute of Biocybernetics and Biomedical Engineering, Polish Academy of Sciences, Ksiecia Trojdena 4, 02-109 Warsaw, Poland; cwojciechowski@ibib.waw.pl (C.W.); achwoj@ibib.waw.pl (A.C.)

**Keywords:** polyethersulfone, tissue engineering, medicine, nanofibres, scaffolds, membranes, biopolymers, hollow fiber membrane, biotechnology, microcapsules

## Abstract

Polymers stand out as promising materials extensively employed in biomedicine and biotechnology. Their versatile applications owe much to the field of tissue engineering, which seamlessly integrates materials engineering with medical science. In medicine, biomaterials serve as prototypes for organ development and as implants or scaffolds to facilitate body regeneration. With the growing demand for innovative solutions, synthetic and hybrid polymer materials, such as polyethersulfone, are gaining traction. This article offers a concise characterization of polyethersulfone followed by an exploration of its diverse applications in medical and biotechnological realms. It concludes by summarizing the significant roles of polyethersulfone in advancing both medicine and biotechnology, as outlined in the accompanying table.

## 1. Introduction

Polymers are used in various areas of technology and science research like biotechnology, chemical, water purification, the drink and food service industry, medical applications, or tissue engineering (TE) [1,2,3,4,5,6,7,8,9,10]. TE is a science that combines biotechnology, medicine, and material engineering. Therefore, TE offers new alternatives for tissue repair and treatment via transplantation of biological material that would replace, maintain, or restore the function of damaged tissues or even whole organs [11,12,13,14,15,16]. TE opens up new avenues for promising therapies. Depending on the application, the usage material should have appropriate properties such as biocompatibility, appropriate structure, or even bioresorbability [17,18,19,20]. The common materials used for TE are natural, synthetic polymers, or their combination (hybrid material) [12,20,21,22,23,24,25]. 

Among the common and frequently used synthetic polymers in medicine are polyesters such as polylactide (PLA), polycaprolactone (PCL), or their copolymers like poly(L-lactide-co-ε-caprolactone) (PLCA). The polymers mentioned are biocompatible and can degrade into non-toxic products that are easily removed from the body [21,26,27,28,29,30,31,32,33,34,35,36]. There are more mentions of non-biodegradable synthetic polymers. They are used not only in industry but also in TE and in many fields of medicine. These include polyethersulfone (PES) biomaterial with good biochemical properties such as strength and biocompatibility. It is often used to obtain hybrid materials or blends. PES is used in many medical areas and biotechnology. Due to their properties, synthetic polymers can be used in various forms, including membranes/scaffolds, capsules, hydrogel, sponges, or nonwovens [26,37,38,39,40,41,42].

The article presents the characteristics of PES polymers along with their use in medicine and biotechnology. In addition, the possibility of its modification in the direction of obtaining components and hybrid materials with prospective applications in medicine will be presented.

## 2. Characteristics of PES

**Figure 1:**
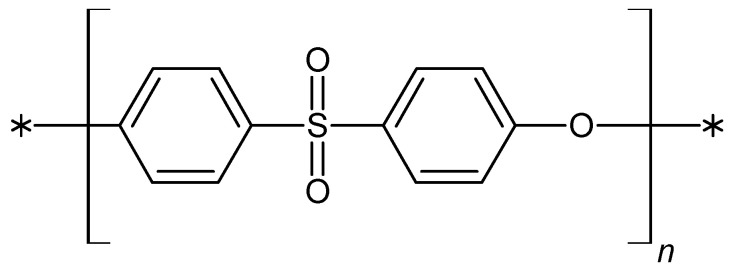
The structural formula of PES, where n = 50–80.

PES (Figure 1) is a linear amorphous polymer. It contains a sulfone [-SO_2_] group that provides adequate stiffness, ether bonds [-O-] that ensure appropriate flexibility, and aromatic rings that contribute to chemical and thermal stability. PES shows good chemical, physics, and biological properties like resistance to many organic solvents, UV, or ionizing radiation. It is characterized by good mechanical resistance, poor water absorption, and thermal strength in the temperature range of 180–220 °C [30,41,43,44,45].

Water absorption is an important property to consider when evaluating the performance of polymers, especially in biomedical applications where contact with bodily fluids or water is common. Therefore, a comparison of water absorption values for PES with other biocompatible polymers such as polyvinylidene fluoride (PVDF), PLA, and PCL has been conducted. The water absorption of PES is low, approximately 0.3% [46,47]. The water absorption for PVDF is lower than for PES, around 0.1%. PVDF exhibits good resistance to water absorption, contributing to its durability and stability in various environments. It is commonly used in biomedical applications such as membranes for filtration and drug delivery systems [48]. PLA has higher water absorption compared to PES material, approximately 1.0%. PLA is a biodegradable polymer derived from renewable resources such as corn starch or sugarcane. Its water absorption properties make it suitable for applications such as biomedical implants, sutures, and drug delivery systems. However, excessive water absorption can affect its mechanical properties over time [49]. The highest water absorption among the mentioned biomaterials is observed in PCL, approximately 2.0%. PCL, like PLA, is a biodegradable polyester with good biocompatibility and flexibility. Its water absorption properties make it suitable for applications such as tissue engineering scaffolds, drug delivery systems, and wound dressings. However, the high water absorption may lead to swelling and degradation over time in aqueous environments [50]. All these polymers are biocompatible and find applications in various biomedical fields; however, their water absorption properties vary. PES and PVDF typically exhibit lower water absorption, while PLA and PCL may absorb more water, which can influence their performance in different applications. The choice of polymer depends on the specific requirements of the application, including mechanical strength, biocompatibility, and resistance to water or other environmental factors. Due to its low water absorption, PES material is often successfully modified to enable its promising use in biological research. In recent years, numerous studies have focused on enhancing the PES material through modifications aimed at improving its water absorption properties, such as blending it with other materials, applying coatings of gelatin or collagen, or introducing amide groups via plasma treatment [41,51,52,53,54].

PES is known for its excellent chemical stability, which makes it suitable for various applications in industries ranging from healthcare to electronics, so it is a valuable material in many fields of science [55,56,57,58,59,60].

The Solvent Compatibility of PES is high. It is resistant to a wide range of organic solvents, acids, bases, and common chemicals. It is generally stable in polar solvents such as alcohols, ketones, and esters, as well as non-polar solvents like hydrocarbons. Solvents like chlorinated hydrocarbons and strong oxidizing agents may have some effect on PES, potentially causing swelling or degradation over prolonged exposure. However, compared to many other polymers, PES maintains its structural integrity well in the presence of various solvents [30,41,43,44,45].

Photosensitivity: PES is relatively stable to ultraviolet (UV) radiation and sunlight exposure. It does not undergo significant degradation or discoloration upon prolonged exposure to sunlight, which is advantageous for outdoor applications and devices ex-posed to light. However, like most polymers, prolonged exposure to UV radiation can still lead to some degree of degradation, primarily surface degradation or changes in mechanical properties. In addition, PES products can be easily sterilized using the following methods: thermal, steam-thermal, ethylene oxide, ethylene alcohol, or γ or β ionizing radiation. The mentioned techniques do not affect the degradation of PES, which is crucial for medical device applications. Furthermore, it can be obtained in a non-toxic and biocompatible form that is very important in the biomedical application [30,41,43,44,45].

PES membranes are extensively used in medical fields: a wide range of medical equipment is used for blood purification devices (hemodialysis, hemofiltration, plasmapheresis, hemodiafiltration, and plasma collectors) and even to obtain artificial organs trough culture on scaffolds various cells [30,41,43,44,61,62].

Nonetheless, upon exposure to blood, proteins quickly adhere to the PES membrane’s surface, forming a protein layer that can result in unfavorable outcomes, including platelet adhesion, aggregation, and coagulation. Consequently, the PES membrane’s blood compatibility is insufficient, necessitating the use of anticoagulants during clinical applications. Additionally, the PES membrane is water-stable and inert, serving solely as a separation barrier, making it ill-suited for advanced separation applications like intelligent separation [51,63].

PES has found extensive applications in both medical uses and wastewater treatment membranes due to its outstanding mechanical and thermal characteristics. However, a primary drawback of PES is its almost hydrophobic nature, which can lead to biofouling issues and has long been considered a significant limitation in membrane technology. Biofouling refers to the accumulation and growth of microorganisms, such as bacteria, algae, and marine organisms, on the surfaces of various structures, materials, or equipment submerged in water. This unwanted biological colonization can lead to deterioration, reduced performance, or contamination of the affected surfaces [64]. To address this concern, chemical modification of the PES material emerges as a promising solution. This study also examines previous research efforts that have explored the incorporation of amino-functionalized PES with various nanomaterials to promote biological activity and mitigate fouling problems in the manufactured membrane. The introduction of hydrophilic functional groups via surface modification is widely recognized as one of the most commonly employed strategies to enhance the hydrophilicity of PES while preserving its mechanical and thermal properties [45,65,66,67,68,69,70].

## 3. The Application of PES in Biotechnology and Medicine

**Figure 2:**
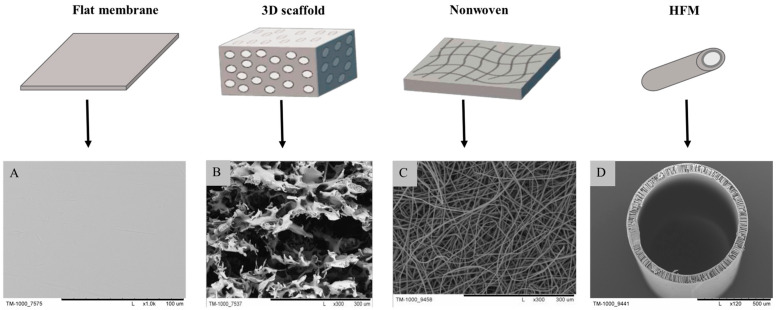
Schematic illustrations of the primary forms of PES polymer utilized in medicine and biotechnology, along with their photomicrographs (**A**–**D**). Scale bars: (**A**) 100 µm; (**B**,**C**) 300 µm; (**D**) 500 µm.

PES material is used in many medical fields. It is most commonly found in the form of hollow fiber membranes (HFMs), nanofibers, three-dimensional and flat membranes, and others (Figure 2).

The applications of PES components in biotechnology and medicine have been listed below. 

### 3.1. Scaffolds 3D

Three-dimensional scaffolds (Figure 2) made of PES are primarily used for cell culture purposes. It is necessary for their structure to closely resemble the extracellular matrix (ECM) of the respective cells. They provide the appropriate environment for cell growth, which also determines tissue regeneration appropriately. To support the cell culture, scaffolds need to create a suitable milieu that facilitates cell attachment, movement, and growth. This is achieved through the presence of a suitable structure design, controlled degradability, proper mechanical characteristics, and biocompatibility. Numerous structural attributes, such as porosity, pore size, interconnectedness, and permeability, significantly influence the development of new tissue and the process of regeneration [17,18,19,26,71]. The literature reports the present use of 3D scaffolds for the regeneration of, among others, cartilage and liver.

Articular cartilage, due to its lack of vascularization and innervation, does not have the capacity for self-regeneration. It is an essential component for the proper functioning of the musculoskeletal system [72,73,74,75]. In the literature, there are reports regarding the use of 3D PES scaffolds for the regeneration of hyaline cartilage.

**Figure 3:**
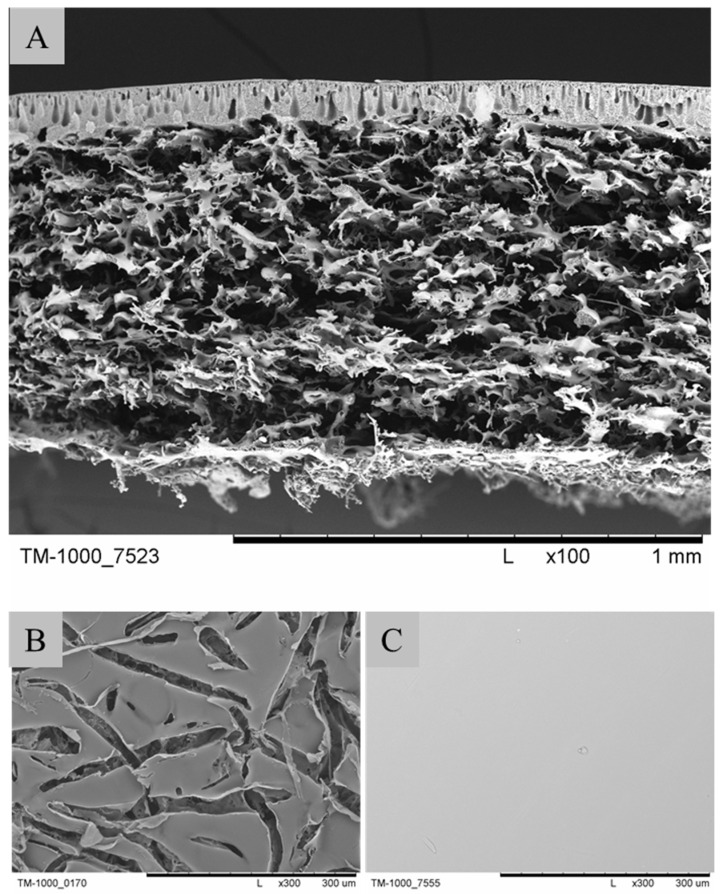
SEM images of PES scaffolds, which were used for in vitro and in vivo research: (**A**) cross- section of scaffold; (**B**) top layer of scaffold; (**C**) bottom layer of scaffold. Scale bars: (**A**) 1 mm; (**B**,**C**) 300 µm. Pictures were prepared according to the literature [30].

The PES scaffold (Figure 3) has undergone preclinical testing on an animal model (rabbit). The method presented by Płończak and colleagues for the culture and transplantation of chondrocytes on PES membranes led to the growth of regenerated tissue, displaying a morphology similar to hyaline cartilage. The research was compared with the use of a commercial scaffold, Chondro Guide (collagen type I/III), and similar outcomes were observed. Furthermore, in histological examinations at the regeneration site, no membranes were observed after 52 weeks. This indicates their resorption in a living organism. It was also mentioned that the excellent results of PES membranes are indicative of the presence of sulfonic groups, which are naturally found in the chondroitin sulfate present in joint cartilage [43]. 

Three-dimensional PES membranes were also subjected to in vitro studies using human chondrocytes isolated from post-operative waste. The results were compared to PLA membranes where the PES membrane outperformed [30]. The membrane exhibited the appropriate structure and strength, which are essential for chondrocyte cultivation. The membrane cross-section features a network of interconnected macropores (Figure 3A). The top surface is porous, allowing cells to penetrate the scaffold’s interior (Figure 3B). In contrast, the bottom is compact, preventing cell escape (Figure 3C) [26,30,43].

**Figure 4:**
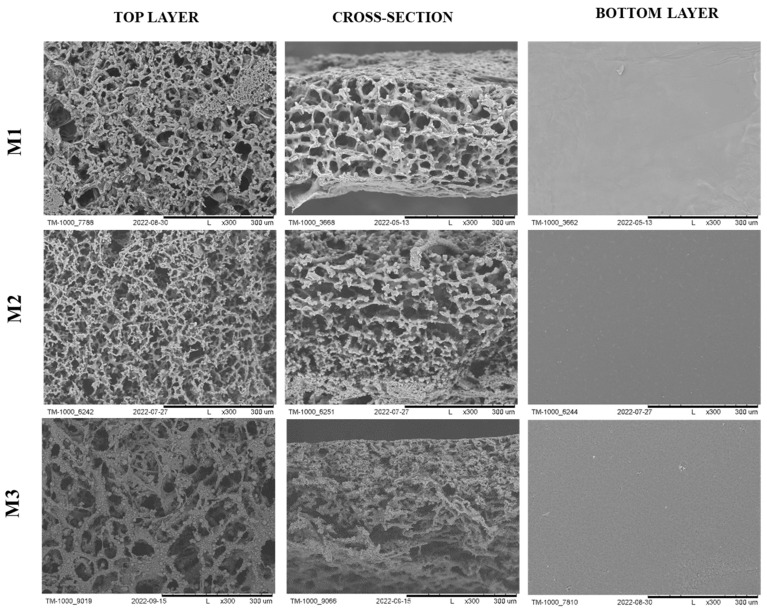
The SEM photomicrographs of the M1–M3 scaffolds. Scale bar: 300 µm. The figure was modified according to the literature [42].

Wasyłeczko and colleagues also obtained scaffolds made from a mixture of PES polymer and biodegradable polyurethane (PUR), which were synthesized [76,77,78]. The membrane structure shows great promise for cartilage tissue engineering. In their studies, they conducted degradation tests of the scaffolds in simulated body fluid, resulting in a mass loss for both polymers. Scaffolds M1–M3 (Figure 4) were obtained by combining the wet phase inversion and salt leaching methods using different polymer weight ratios of PES:PUR where M1 was 1:1, M2 was 2:1, and M3 was 1:2. As the macropore generators, gelatin nonwovens and NaCl salt were used. Unfortunately, membranes have not yet undergone testing with cells or animal models [42].

The liver, in contrast to cartilage, has a high potential for regeneration and rebuilding of damaged tissue. Impaired regeneration contributes to fibrosis or cirrhosis of the liver. Liver impairment can result from exposure to strong toxins like alcohol, substances, medications, hepatotoxic viruses, or even an inadequate diet (e.g., excess salt). To facilitate its regeneration, techniques like cell transplantation (hepatocytes and stem cells) are utilized, and they proliferate, among other methods, within 3D scaffolds, HFMs, or nonwovens/nanofibers [79,80,81,82].

Kinasiewicz and colleagues evaluated spongy PES membranes as a synthetic substrate for cultivating hepatic cells. The bio-implants were transplanted into SCID/NOD mice. It was observed that the PES scaffold was well tolerated by C3A cells. Furthermore, no toxic effects were observed during the study. Collagen gel was used as a medium to support the cells on the scaffold, providing an opportunity to increase the number of cells seeded on the membrane. This research demonstrated that the incorporation of collagen gels with PES scaffolds enables the creation of bioartificial organs with a substantial cell density suitable for transplantation [83].

In another study, it was demonstrated that C3A cells thrive on PES membranes, forming microvilli characteristic of healthy hepatocytes. The cells displayed a strong affinity for PES membranes, adhering to nearly 90% within the initial 24 h of incubation. It was observed that albumin production in the culture increased. The research has confirmed that PES membranes can be used as scaffolds for hepatocyte cultivation [84]. 

### 3.2. Hollow Fiber Membranes

HFMs membranes (Figure 2) are thin structures with a porous construction that can separate various substances (filtration application) based on their size and chemical properties. These kinds of membranes are particularly useful in biotechnology, medicine, and various other fields. The production of capillary membranes can be a technological process. The process of producing HFMs is called “spinning capillary membranes”, and it is a technique commonly used to manufacture micro- and ultrafiltration membranes with microscopic or nanoscale pores [76,77,85].

In biotechnology and medicine, HFMs, made of PES material, are used not only for blood purification from toxins (dialysis) but also as a potential material for bioartificial liver tissues, pancreatic islet transplantation, or the culture of other cells [86,87]. The HFMs are placed in suitable modules before application, which are placed in dialyzers or bioreactors [85,88].

Hemodialysis is a medical procedure used to purify the blood of individuals whose kidneys do not function properly. It is one of the main methods for treating chronic kidney failure. The procedure involves passing the patient’s blood through a special device called a dialyzer or artificial kidney to remove toxins, excess fluids, and electrolytes from the body. Hemodialysis is often performed in specialized dialysis centers, but it can also be performed at home by trained patients, especially in the case of home hemodialysis. This procedure helps maintain electrolyte balance and removes toxins from the body, functions that healthy kidneys typically perform. Hemodialysis is an effective method for keeping patients alive in cases of advanced kidney failure [44,89,90,91].

PES high-flux membranes are frequently employed in dialysis machines and represent one of the common membrane types used in these devices. PES membranes possess properties that render them well-suited for renal dialysis applications, including excellent permeability, mechanical strength, and high toxin removal efficiency. Consequently, PESmembranes are a popular choice in modern hemodialysis dialyzers, helping to guarantee an effective and safe blood purification procedure for patients with renal failure. Due to the hydrophobic nature of the PES material, problems can arise during hemodialysis in the form of biofouling (adsorption of proteins and other substances from the patient’s blood), reducing the effectiveness of the dialysis procedure. However, some various methods and strategies help address this issue [37,41,45,51,61,67,68].

Xin Zheng and colleagues obtained HFMs consisting of PES and a hydrophilic ionic liquid (IL) that was prepared using a dry-wet spinning method. A comprehensive analysis was conducted to study the configuration and characteristics of PES-g-IL HFMs, which included aspects such as hemocompatibility, hemodialysis effectiveness, and cytotoxicity. A comparison of PES-g-IL HFMs was made with the PES membrane, resulting in a significant improvement in the material with the addition of IL. This study provided a new approach for creating and developing high-performance hemodialysis membrane materials [92].

Examples of commercial HFMs made from PES are Purema [93,94,95] and ELISIO [95,96].

Another membrane that has been designed for hemodialysis using PES material with amounts of 2 wt% SlipSkin^TM^ (SS) was PES-SS2. The SS additive is a copolymer consisting of hydrophilic N-vinylpyrrolidone (NVP) and hydrophobic N-butylmethacrylate (BMA) that changes the properties of the membrane. The results indicate that the membranes exhibit significant resistance to fouling by proteins and intermediate-sized molecules, in addition to displaying excellent blood compatibility. The inclusion of SS enhances the potential of these membranes for use in dialysis therapy. Subsequent research will prioritize a thorough examination of their effectiveness in filtering uremic toxins from both human plasma and whole blood. The membrane was comparable to benchmark membranes [97].

Hollow fiber bioreactors find extensive application in bioartificial livers. These instruments comprise a collection of PES semi-permeable capillary membranes that replicate the natural vascularization found in liver tissue. Such devices, with their substantial surface area, facilitate efficient two-way mass transfer [82]. 

Verma and colleagues obtained PES–carbon nanotubes composite HFMs for a bioartificial liver. Membranes were fabricated via the phase inversion method using sing d-α-tocopheryl polyethylene glycol 1000 succinate (compatibilizer) and carboxylated multiwalled CNTs (filler), which was optimized for improved hemocompatibility, cellular functionality, and cell viability. PTC-2 membrane with a concentration of 0.030 wt.% filler proved to be the most favorable option. It showed improved compatibility with human blood. The biocompatibility of these membranes underwent testing with human liver cell lines (HepG2). The research revealed heightened liver cell proliferation and enhanced functionality, particularly in terms of albumin secretion and glucose consumption. These composite HFMs, with their 3D scaffold-like characteristics for cell growth and selective permeability that aids immunoisolation, hold potential for use in liver cell bioreactors and the development of bioartificial livers [98]. 

**Figure 5:**
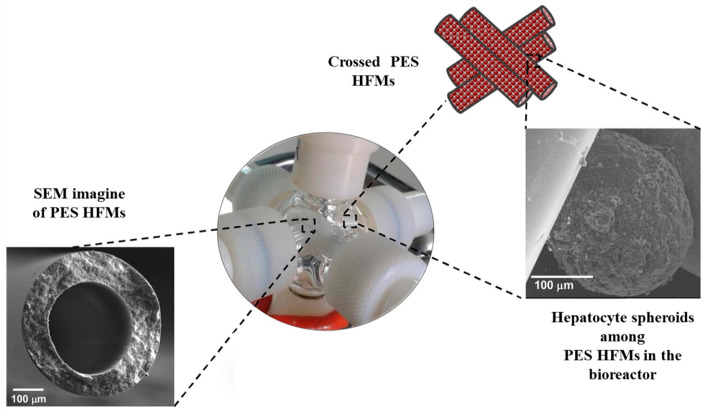
Schematic illustration of a 3D liver microtissue model. The picture was modified according to the literature [99].

In another study, a three-dimensional liver tissue model was developed using spheroids of human hepatocytes (cryopreserved primary human hepatocytes isolated from human tissue), which were cultured in a bioreactor with a PES membrane made of intersecting HFMs (Figure 5). These spheroids fused to form larger structures resembling microtissue, distributed around and between the PES membranes. The study demonstrated that the microtissue spheroids successfully maintained their viability and functionality for 25 days. Urea synthesis, albumin secretion, and diazepam transformation were observed. The bioreactor provided a well-controlled microenvironment at the molecular level due to the selective permeability properties of the membranes and the dynamic fluid flow conditions, enabling continuous delivery of nutrients and removal of catabolites and specific cellular products. Sufficient oxygen supply was also ensured, as confirmed via experimental measurements of oxygen concentration and oxygen consumption rates by the cells. Oxygen concentration profiles in both the spheroids and the extra capillary space confirmed the bioreactor’s ability to provide adequate oxygenation to the microtissues. The obtained bioreactor, utilizing spheroids of human hepatocytes, has the potential to be used in studies related to drug toxicity and liver diseases that require long-term cultivation [99].

Unger et al.’s research revealed that human endothelial cell growth was facilitated by porous PES HFMs. Freshly isolated human dermal endothelial cells (HDMECs), pulmonary endothelial cells (HPMECs), umbilical vein endothelial cells (HUVECs), as well as two human endothelial cell lines, HPMEC-ST1.6R and ISO-HAS.c1, were introduced onto PES HFMs. Prior coating of PES with gelatin or fibronectin was necessary for cell adhesion and spreading on the uneven, porous surface over time. Confluent cells exhibited typical strong PECAM-1 expression at cell–cell boundaries. The cell adhesion and growth were monitored using confocal laser scanning microscopy. Slight expression of activation markers E-selectin, *ICAM-1*, and *VCAM-1* was observed via the real-time polymerase chain reaction (RT-PCR) in cultured cells. However, after 4 h of LPS stimulation, activation of these markers was observed, and immunofluorescent staining confirmed induction in the majority of cells, thus confirming their intact functionality [100].

Endothelial cells grown as monolayers on PES migrated, forming structures resembling microvessels when placed in angiogenesis-stimulating conditions. Therefore, human endothelial cells cultured on fibronectin-coated PES HFMs retain important morphological and functional characteristics specific to endothelial cells. Studies show that PES HFMs can serve as a valuable biomaterial in biotechnological and tissue engineering applications [100].

The pancreas is an organ located in the abdominal cavity that is an important part of the digestive and endocrine systems. It consists of several different components, including pancreatic islets. Pancreatic islets are small groups of cells that play a crucial role in producing hormones such as insulin and glucagon. There are two main types of pancreatic islets: Langerhans islets (groups of cells in the pancreas responsible for hormone production) and Exocrine islets (type of islets in the pancreas responsible for producing digestive enzymes, responsible for digestion and absorption of nutrients from food). Pancreatic islets are extremely important for regulating blood glucose levels and the functioning of the digestive system. Damage to pancreatic islets or their improper function can lead to diseases such as diabetes, characterized by disturbances in glucose metabolism and insulin resistance. Pancreatic diseases can be serious and require specialized medical treatment and care [101,102]. 

Diabetes is a metabolic disorder that affects blood sugar levels. There are different types of diabetes, but the most common ones are type 1 diabetes (insulin-dependent), type 2 diabetes (insulin-resistant), and gestational diabetes. Type 1 diabetes is an autoimmune disease in which the immune system attacks and destroys the beta cells in the Langerhans islets, which are responsible for producing insulin. Individuals with this disease must regularly take insulin because they cannot produce this hormone. It is typically diagnosed in young people and requires daily monitoring of blood glucose levels and insulin dosage control. Type 2 diabetes is the most common type of diabetes. In this case, the body’s cells become resistant to the effects of insulin, and the pancreas is unable to supply sufficient insulin to maintain normal blood sugar levels. It is often associated with risk factors such as obesity, lack of physical activity, diet, and genetics. Managing type 2 diabetes often involves lifestyle changes, diet, physical activity, and oral medications or insulin in some cases. Untreated or poorly managed diabetes can lead to problems such as damage to blood vessels, neuropathy, heart diseases, kidney damage, and vision issues. Proper treatment and medical care are essential for individuals with diabetes [103].

The HFMs made of PES are also utilized in pancreatic applications. The study was specifically designed to evaluate a flat immunoprotective device for the subcutaneous xenotransplantation of pancreatic islets in diabetic rats. The device is constructed using parallel arrays of HFMs. The in vitro permeability properties and the in vivo performance of three distinct PES HFMs with varying membrane ultrastructures were compared. In all diabetic rats, the implantation of devices containing islets rapidly normalized hyperglycemia. The in vitro membrane permeability to glucose was correlated with the implant’s performance. As a control condition to assess islet function, bovine islets enclosed in alginate gel were implanted into the peritoneal cavity of diabetic rats. These results demonstrate, for the first time, the feasibility of using the new immunoisolation device for subcutaneous xenotransplantation of islets, resulting in transient normalization of hyperglycemia in immunocompetent diabetic rats. Prevascularization of the subcutaneous site may provide a more favorable microenvironment, prevent islet necrosis, and extend the functional duration of this artificial pancreas [104].

An alternative to traditional animal research is the creation of organoids in laboratories, aiming to reduce the need for animal experimentation. Organoids are three-dimensional cell cultures that serve as miniaturized models of organs or tissues. They are laboratory-grown cellular structures that possess features and functions similar to real organs or tissues. They are used in scientific research, especially in the fields of biology and medicine, for studying developmental processes and diseases, drug testing, and assessing the side effects of potential therapies. Organoids can be grown from stem cells or cells derived from specific tissues or organs, allowing the creation of experimental models for the study of various biological processes. Organoids are an important tool in biomedical research, enabling scientists to gain a more precise understanding of the biology of the human body [105,106].

Intestinal and intestinal villus organoids serve as a significant research model with numerous applications in biological sciences. They can be used, among other things, to model intestinal functions (studying digestive processes, nutrient absorption, intestinal motility, and similar) and research intestinal diseases such as Crohn’s disease, ulcerative colitis, and others. This enables the examination of the mechanisms underlying these diseases and the testing of new therapies. These organoids allow for toxicity testing of chemical substances and drugs in a model intestinal system, aiding in the assessment of potential risks and side effects. They can also be employed as a model to study interactions between gut microbiota and intestinal cells, which is crucial in the context of research on the gut microbiome and its impact on health. Therefore, these organoids are a valuable research model for studying intestinal function and gastrointestinal diseases. They provide valuable insights into physiological processes and the development of new therapies [107,108,109]. 

HFMs made from PES material were tested to produce intestinal organoids. As part of the research, an intestinal model was created, taking into account physiological parameters such as ECM components and shear stress. Caco-2 cell culture was conducted in a three-dimensional environment, and it was observed that the addition of ECM coating on PES HFMs had a positive impact on the phenotype and morphology of Caco-2 cells. This resulted in the formation of differentiated intestinal tubules. Various types of cells were noted, including enterocytes (confirmed by ALP activity), Paneth cells, goblet cells, enteroendocrine cells, stem cells, and structures resembling villi. Furthermore, experiments were conducted using this model (HFMs with ECM) to investigate the response to toxin A produced by *Clostridium difficile*, which is the main cause of healthcare-associated diarrhea [109].

The research demonstrated that combining PES HFMs with ECM components in a single model holds promise for the development of new drugs, nutraceuticals, food ingredients, and toxin screening studies. Such a combination will expedite the process of polarization and differentiation of *Caco-2 cells*, allowing for the creation of a model that better mimics intestinal physiology. Additionally, the model can be valuable for studying the effects of severe intestinal barrier disorders, such as the action of toxin A produced by *Clostridium difficile* [109].

Nervous tissue is a type of tissue that includes the brain, spinal cord, and peripheral nerves. It is a complex cellular system consisting mainly of neurons and glial cells. It is crucial for the functioning of the nervous system, which controls various aspects of the body’s activities. Disorders in this tissue, such as neuronal damage or loss of myelin sheaths, can lead to various neurodegenerative diseases. They impact the brain’s ability to control movements, memory, and other cognitive functions. Some of the most common neurodegenerative diseases include Alzheimer’s disease (AD), Parkinson’s disease, or Amyotrophic Lateral Sclerosis. Research on nervous tissue is essential for understanding the functions of the nervous system and developing treatment methods for various neurological disorders. Regeneration of nervous tissue is a process that is limited compared to the regeneration of some other tissues in the body. The adult human nervous system has limited regenerative capabilities, primarily due to the lack of cell division in neurons, difficulties in rebuilding synaptic connections, and the presence of the blood–brain barrier, which hinders access to the damaged areas of the brain and spinal cord. Although regeneration of nervous tissue is still an area of intensive research, progress is gradually being made, and scientists are gaining more knowledge about regenerative mechanisms. However, achieving full and effective regeneration of nervous tissue remains a challenge, and the treatment of neurological disorders often focuses on alleviating symptoms and slowing disease progression [110,111,112].

**Figure 6:**
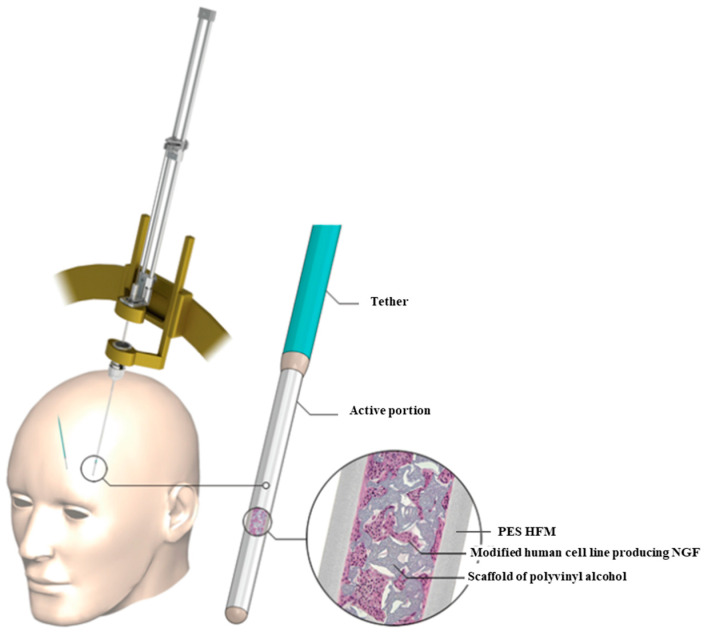
Schematic (**left**) showing the process involved in implanting the left-sided device and Implant NsG0202 (NsGene A/S) (**right**). The figure was modified according to the literature [113].

In the context of nervous system regeneration, capillary membranes made of PES can be used to create supportive structures that facilitate the growth of nerve cells and contribute to the regeneration of damaged areas. Wahlberg and colleagues developed an implant constructed with a HFM made of PES. Clinical studies have been conducted utilizing a device designed for delivering nerve growth factor (NGF) to the basal forebrain. The objective of the research was to impede neuronal degeneration and enhance cognitive functions in patients with AD. In the context of neurodegenerative disorders, targeted delivery of regenerative proteins may demonstrate favorable effects. With further technological advancement, this approach has the potential to become a novel therapeutic tool in the realm of functional and regenerative neurosurgery. The amalgamation of gene therapy benefits with the simplicity and safety of an implantable and retrievable device opens prospects for the continued development of this field. Implant NsG0202 (NsGene A/S) comprises a genetically modified human cell line producing NGF, cultivated on a porous scaffold of polyvinyl alcohol within a semi-permeable HFMs PES membrane (with an outer diameter of 0.72 mm and an average molecular weight of 280 kD). This membrane facilitates the influx of nutrients and efflux of the therapeutic NGF factor. The membrane, in turn, is connected to a neutral polyurethane band, which is ultimately attached at the opposite end to the edge of the opening (Figure 6). Implants were manufactured in accordance with good manufacturing practices, undergoing sterility tests and demonstrating no cell leakage. A 5-week shelf life and 3-day storage in a thermally insulated container at the study site were verified for device viability and NGF production. The primary objectives, including demonstrating implantation feasibility, retrieval capability, 12-month NGF secretion, long-term safety, and tolerance of NsG0202 implants in AD patients, were achieved. With certain adjustments, this neurosurgical rebuilding technological platform justifies additional testing in AD and other significant neurological disorders, where targeted delivery of regenerative proteins may prove beneficial. With further development, this technology has the potential to become a novel therapeutic tool in functional and regenerative neurosurgery, combining the advantages of gene therapy with the simplicity and safety of an implantable and retrievable device [113].

**Figure 7:**
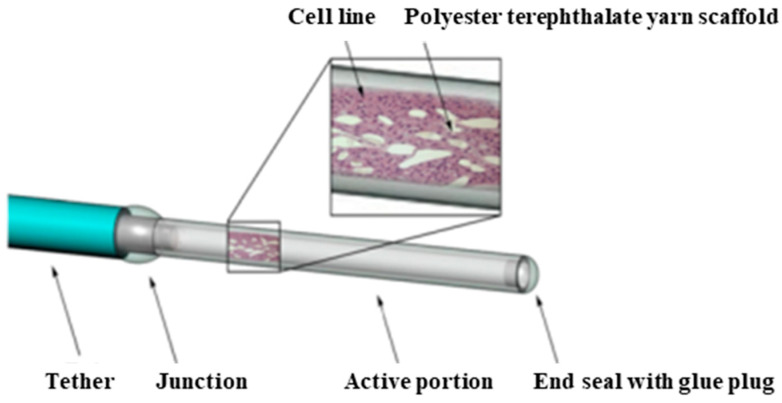
The schema of the second-generation implant, NsG0202.1. The figure was modified according to the literature [114].

The second-generation implant, NsG0202.1, is also designed to deliver NGF as a potential therapy for AD. It has undergone clinical trials in patients with mild to moderate disease over a period of 6 months. The secretion of NGF via second-generation implants has been enhanced through the utilization of Sleeping Beauty transposon gene expression technology and improved three-dimensional internal scaffolding, resulting in the production of approximately 10 ng of NGF per device per day. All patients underwent successful implantation procedures without complications and completed the study, including the removal of the implant after 6 months. Implant NsG0202.1 was manufactured under good manufacturing practices (GMPs). A hollow, impregnated polyurethane strip was attached to a semi-permeable PES HFM with an outer diameter of 0.72 mm and an average molecular weight of 280 kDa. The sponge matrix made of polyvinyl alcohol used in the original NsG0202 device was replaced with a matrix made of polyethylene terephthalate yarn as the internal cell-supporting scaffold (Figure 7). This matrix allowed for better cell adhesion, improved cell survival, and the potential for enhanced production. These data support the ongoing development of the technology for the treatment of AD and other neurodegenerative disorders [114].

### 3.3. Microcapsules 

**Figure 8:**
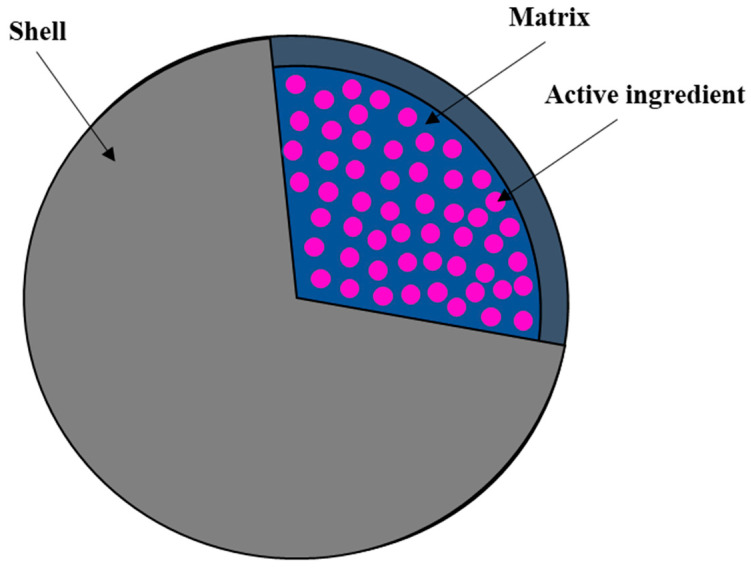
Schematic ilustration of microcapsules.

Microcapsules are structures composed of a substance surrounded by a thin layer or membrane (Figure 8). These structures are designed to protect the contents of the microcapsule and/or control the release of this content under specific conditions. They find applications in various fields of medicine and biotechnology due to their unique properties. They are used, among other things, for controlled drug delivery (drug delivery system, DDS), as carriers for therapeutic gene delivery (treatment of genetic diseases, cancers, and tissue regeneration), in implantology, for transporting biological substances (protecting biological factors from environmental conditions), in diagnostics (carriers of contrast agents), and also in tissue engineering as cell carriers. Thus, microcapsules offer a wide range of applications, and their versatility makes them the subject of intense research in the context of innovative medical and biotechnological solutions [115,116,117].

PESfinds application in this field as well. It is utilized for various purposes, leveraging its advantageous properties. PES serves as a material in areas such as drug delivery, cell carriers, or gene therapy. The utilization of PES in these applications underscores its significance in advancing technologies within the medical and biotechnology domains.

Tan and his team have designed a system comprising two different docking units: one made of polyamide (PAAM) and the other of polyacrylic acid (PAAC). Both materials were, respectively, grafted onto PES microcapsules (designated as PES-g-PAAC or PES-g-PAAM), utilizing intermolecular hydrogen bonding in an aqueous solution. As part of the study, vitamin B12 (VB12) and vancomycin hydrochloride were chosen as the released drugs. Both substances are water-soluble, do not react with each other, and have non-interfering maximum UV wavelengths for measuring the cumulative drug solubility. The results of the release of active substances demonstrate that the docking system is effective and responsive to temperature changes, especially when the grafting ratio of PES-g-PAAM to PES-g-PAAC is close to 1:1. It was observed that, below 25 °C, the system exhibits a “switch-off” effect as polymer chains on the surface of the microcapsule form intermolecular hydrogen bonds. Conversely, at temperatures above 25 °C, hydrogen bonds between the polymer chains of PAAC and PAAM break. Two microcapsules separate, allowing drug molecules to easily penetrate the surface of PES microcapsules, resulting in an “on” state [118].

Drug release experiments are used to verify the response of the drug delivery system to temperature variations and to investigate the quantitative relationship between the speed of microcapsule grafting. The obtained results provide valuable insights for improving the feasibility of multi-drug delivery systems [118].

Kupikowska-Stobba et al. examined the release of human albumin (HA) from microcapsules composed of alginate–polyethersulfone (Alg/PES) using the electrostatic coextrusion method. The PES component forms a hydrophobic, non-biodegradable wall, resulting in a gradual release of HA without a burst effect. The release is solely dependent on the diffusion of the protein desorbed from the Alg hydrogel in the microcapsule core through the membrane pores. The release of HA adhered to first-order kinetics, exhibiting the highest release rate within the initial 5 h and reaching a plateau after 19 h. However, the study also indicates that the actual concentration of released HA from the microcapsules was 60% of the theoretical value for microcapsules with 100% permeability. This discrepancy may be attributed to protein adsorption to the Alg core or PES membrane [119,120].

### 3.4. Flat Membranes

Flat membranes (Figure 2) can be obtained from various materials, including synthetic polymers such as PES, typically using the phase inversion method. They play a crucial role in various fields, including medicine and biotechnology. They are widely used for the filtration of different substances, such as bacteria, viruses, proteins, and other particles. This application is found in the purification of medical fluids like blood and infusion fluids, as well as in the purification processes of proteins and other biotechnological products. They are utilized, among other purposes, in hemodialysis, bioreactors (separation of cells, proteins, or other substances), and drug delivery systems. Additionally, they find application in cell cultures, serving as a substrate.

Flat membranes are utilized as a substrate in the field of stem cells, both in scientific research and potential therapies. Stem cells are unique cells with the ability to self-renew and differentiate into various specialized cell types. There are two main types of stem cells: embryonic stem cells and somatic stem cells (derived from various organs and tissues of the adult body). Stem cells represent an area of biomedical research, and their application holds enormous potential in the fields of medicine, regenerative therapies, and tissue engineering. However, their application requires further research into controlling differentiation processes and preventing potential complications [121,122].

A study by Mori et al. showed that PES flat membranes covered by fibrinogen (Fg) facilitate stem cell adherence and proliferation while maintaining therapeutic properties. Fg, applied as a coating ECM for PES flat membranes, offers advantages over other options, providing a clinically graded product with stable, cost-effective availability, globally compared to recombinants. Enhanced with fibronectin or collagen, PES further improves cellular affinity. In the study, the cells were seeded on Fg-coated PES (Fg-PES) and were cultured for 2 weeks [123].

In conclusion, the Fg-PES is proved to be a widely available, safe ECM, acting as a versatile scaffold for regenerative medicine. It supports stem cell self-renewal by stabilizing fibroblast growth factor (bFGF) signaling and activating autophagy to suppress cellular senescence. Future investigations will explore effects with patient serum and potential unidentified adverse effects, advancing diverse culture systems for clinical-grade stem cells, including serum-free cultures and automated devices [123]. 

**Figure 9:**
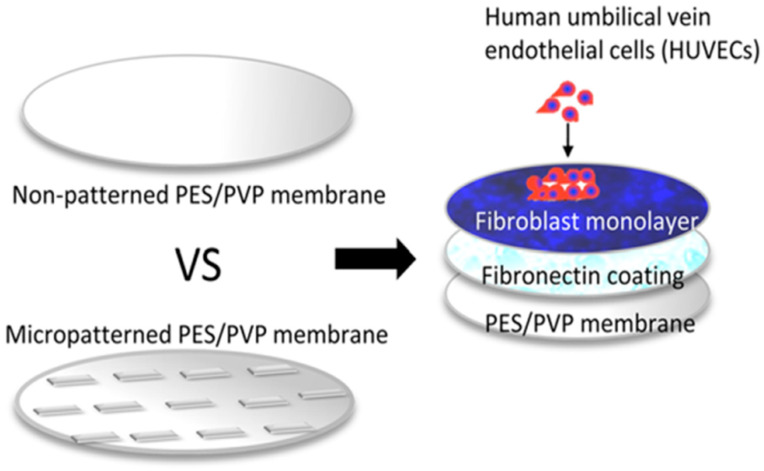
Schema of encapsulation device. Image was prepared according to the literature [120].

One treatment option for patients with type 1 diabetes is islet transplantation, where patients require immunosuppression to prevent rejection. To avoid the need for toxic immunosuppression while simultaneously increasing the chances of graft survival and function, encapsulating islets using flat semi-permeable membranes has proven effective. This approach enables the creation of a barrier against immune cells and cytotoxic molecules. Additionally, this prevents rejection and allows for the diffusion of oxygen, nutrients, and hormones. Skrzypek and colleagues obtained thin semipermeable membranes of polyethersulfone/polyvinylpyrrolidone (PES/PVP) with a micropattern using micro-molding with phase separation (PSμM), where the bottom membrane with microcavities provides good separation of encapsulated islets, and the top flat membrane acts as a cover. The technique used allowed for the production of porous membranes with the desired surface topography in a single step. To achieve cell adhesion properties on one side of the membrane, a thin layer of fibronectin was applied. Human dermal fibroblasts (NHDF) were used for the study as supporting cells in co-culture with human HUVECs (Figure 9). To assess the influence on cell organization and their interconnections in culture, membranes with continuous and interrupted linear patterns (micro-patterned) and non-patterned PES/PVP membranes were used. As a result, it was demonstrated that the micropatterned membrane achieved cell alignment towards the formation of early microvascular networks [124].

**Figure 10:**
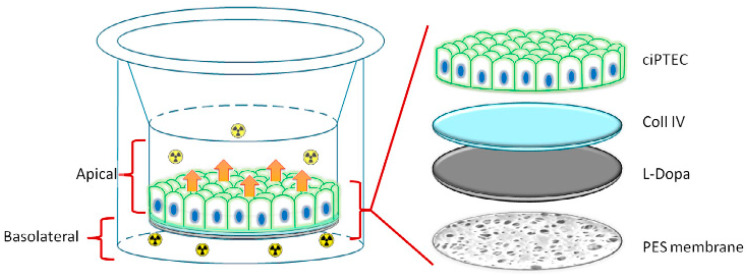
The “living membrane” created by Schophuizen et al. The figure was modified according to the literature [125].

Schophuizen et al. presented promising results in the development of a bioartificial kidney, particularly focusing on the creation of a “living membrane—a crucial component of such a device (Figure 10). In this study, a flat PES membrane was used. The proposed device consists of a flat PES membrane coated with a double layer of 3,4-dihydroxy-L-phenylalanine (L-DOPA) and human collagen IV (Coll IV). To develop a living membrane with a functional layer of proximal tubule epithelial cells (ciPTEC), a unique, conditionally immortalized cell line of ciPTEC was utilized. The PES membrane with a molecular weight cut-off of 50 kDa was used to prevent albumin leakage and block immunoglobulin transfer, thereby preserving essential blood components in case of monolayer cell integrity compromise. The coating of the membrane with L-DOPA and Coll IV was applied to its more porous and rough side to enhance cell adhesion. Additionally, the coating was carefully optimized to maintain membrane permeability to bodily fluids and blood components while creating a tight monolayer of cells. The presented results demonstrated successful development of a living membrane consisting of a repeatable monolayer of ciPTEC on PES membranes, representing a promising advancement in the field of bioartificial kidney development [125].

The study obtained by Yeo et al. involved the fabrication of a PES membrane, which underwent immersion ion implantation in plasma (PIII) to obtain a suitable substrate for immobilizing biomolecules. The aim was to provide a biomimetic material that integrates with tissues and regulates the formation of new tissues. The PIII treatment was utilized to enhance the wettability of PES and induce the formation of acidic groups on the membrane surface. Researchers demonstrated that surfaces subjected to 80 s of PIII exhibited the highest free surface energy and the highest concentration of acidic groups. Subsequently, the membrane surfaces were coated with tropoelastin, an ECM protein, to mimic the natural cellular environment and elicit a desired biological effect. This prepared surface is resistant to molecular contaminants and actively promotes the adhesion and proliferation of various cell types, stimulating specific cellular responses and guiding tissue formation. Tropoelastin was chosen due to its retained bioactivity after ethylene oxide sterilization, as confirmed in conducted studies. This is crucial for devices intended for clinical use [126].

Researchers evaluated the obtained membrane regarding the influence of PIII treatment on its structure, hydrophilicity, ability to covalently bind tropoelastin, and its resistance to ethylene oxide sterilization. Finally, the tropoelastin-modified and functionalized PES membrane was tested for its ability to promote adhesion, proliferation, and maintenance of the morphology of human dermal fibroblasts, demonstrating very promising results in the study [126].

In the next research, Zailani et al. successfully improved the hemocompatibility of PES flat membranes through poly (1,8-octanediol citrate) (POC) blending. The researchers obtained three membranes (M1–M3) based on PES, which were doped with POC at concentrations of 1%, 2%, and 3%, respectively. The purpose of POC was to enhance hemocompatibility. A pore-forming agent, polyvinylpyrrolidone (PVP), was also used in the membrane-forming solution. The resulting compositions were evaluated through analysis of adsorption of human serum Fg, activated partial thromboplastin time (APTT), blood platelet adhesion, and prothrombin time (PT). Additionally, levels of thrombin-antithrombin III (TAT), generation complement (C3a and C5a), and calcium ion (Ca^2+^) absorption on the membrane were examined. The analysis of the results indicated that increasing the concentration of POC significantly reduced FBG adsorption, prolonged APTT and PT, reduced blood platelet adhesion, activated complement factors C5a and C3a, and lowered TAT levels. Furthermore, increased Ca^2+^ absorption was observed. It was demonstrated that the modified membranes exhibited improved hemocompatibility properties with increasing POC content, especially M3. This has significant implications for the hemocompatibility of the material, particularly in the context of blood purification technologies, such as hemodialysis [127,128].

The PES filter membrane represents one of the most significant and widely recognized commercial applications. The PES membranes are used, among others, in internal intravenous filters. They protect against infection by particles, bacteria, fungi, and endotoxins and eliminate air bubbles. The Intrapur^®^ Neonat filter is equipped with a PES Supor^®^ 0.2 µm membrane, which has an effective filtration area of 1.65 cm^2^. It helps prevent contamination by suspended dust by trapping particles larger than >0.2 µm, reduces microbiological contamination by filtering bacteria and fungi, while the positive charge of the membrane aims to trap endotoxins [129]. Another example is the Intrapur^®^ Lipid, a 1.2 µm infusion filter for lipid emulsions. It is equipped with a PES Supor^®^ 1.2 µm membrane, with an effective filtration area of 10 cm^2^. It helps prevent contamination by suspended dust by trapping particles larger than >1.2 µm. It reduces microbiological contamination by filtering fungi, has low protein binding, and allows lipids to pass through [130]. Both of the mentioned PES membranes reduce the risk of medication administration error [129,130].

Another instance showcasing filtration applications is the utilization of PureFlo^®^ Z Series PES capsules. These capsules feature a hydrophilic, asymmetric PES membrane renowned for its remarkable throughput capacity and liquid flow performance. The filter capsules integrate a final sterilizing-grade membrane of either 0.1 or 0.2 µm, along with an optional ZenFlo^®^ highly asymmetric prefilter designed to augment filter efficiency across various biopharmaceutical processes [131,132].

### 3.5. PES Nanofibers

Nanofibers are very thin fibers with nanoscale diameters. Nanofibers play a crucial role in biomedical engineering due to their unique properties and versatile applications. They are used, among others, to create three-dimensional structures resembling human tissues, which can be used in laboratory studies to test drugs, as well as medical implants for tissue regeneration or replacement of damaged tissues; they can be used to produce dressings and wound healing materials. Their large surface area provides excellent permeability to fluids and gases, which promotes the healing process; nanofibers can serve as drug carriers, enabling controlled release of active substances at specific locations and times. They are used in tissue engineering to create growth substrates for cells to reproduce complex tissues and organs, which is important in regenerative therapies and transplantology. They are also implemented as sensors for detecting biomarkers or other substances in the body, where their high surface area and ability to function on a microscale make them ideal for detecting even the smallest amounts of substances. The use of nanofibers in biomedical engineering opens up new therapeutic and diagnostic possibilities, which can contribute to improving the health and quality of life of patients [133,134,135,136,137,138].

The most popular method for producing nanofibers is electrospinning. The process involves using high electrical voltage to draw polymer fluid through a needle, thereby creating thin fibers. This method is relatively simple and allows for control over the size and shape of nanofibers [133,136,137,138].

The chapter presents nanofibers made of PES, which find application in various fields of medicine and biotechnology. Nanofibers, due to their properties, are utilized, among other applications, in the cultivation of osteoblasts. They facilitate the growth and differentiation of bone cells for the purpose of conducting research on bone regeneration, treatment of bone injuries, and development of new implant materials used in orthopedic surgery. Their utilization can accelerate fracture healing processes, reduce the risk of implant rejection, and improve the quality of life for patients with bone diseases and injuries [138]. In the literature, reports can be found regarding the utilization of PES nanofibers for the cultivation of osteoblasts.

Shabani and colleagues fabricated nanofibrous membranes from PES, which were subsequently modified and seeded with unrestricted somatic stem cells (USSCs) to mimic the natural bone structure. The team obtained PES nanofibers without treatment (PES), PES nanofibers treated with plasma (PL-PES), and collagen-grafted PES nanofibers (COL-PES). The resulting scaffolds featured a nanofibrous and highly porous structure with a large surface area. The membranes were characterized physico-chemically, and then their ability to support proliferation, infiltration, and osteogenic differentiation of USSCs into osteolineage cells was evaluated using an induction medium. Surface treatment led to increased hydrophilicity and biocompatibility of the nanofibers. During osteogenic differentiation of stem cells on COL-PES nanofibers, the highest levels of alkaline phosphatase activity and calcium content were observed. RT-PCR showed significant differences in gene expression related to osteoblasts on COL-PES compared to other substrates [139].

Presented COL-PES nanofibers hold potential for bone grafting due to their nanofibrous, three-dimensional structure, and bioactivity. This structure supports proliferation, differentiation, and infiltration of USSCs, as confirmed by assessments of osteogenic markers and histological examinations. COL-PES nanofibers demonstrate the ability to heal and regenerate bone [139].

**Figure 11:**
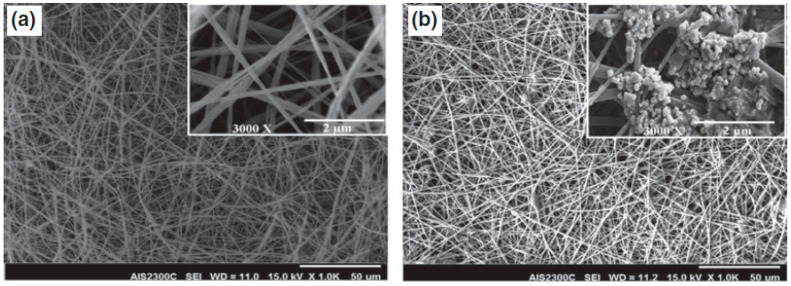
SEM picture of PES nanofibers (**a**) and BG-coated PES (**b**). Scale bar: 50 µm. The picture was modified according to the literature [141].

The next example is modified PES nanofibers, which also find application in bone tissue regeneration. Ardeshirylajimi and colleagues obtained PES nanofibers using the electrospinning method, which were then coated with bioactive glass (BG) (Figure 11). Their ability to support cell proliferation was investigated in vitro using the MG-63 cell line. Cell viability was determined using MTT assay. Osteoconductivity was assessed using common osteogenic markers. Finally, in vivo studies were conducted on a rat model using imaging analysis (digital mammography), computer tomography, and histological examination. In vitro results showed that the biocompatibility, osteogenic markers alkaline phosphatase (ALP) activity, and calcium mineral deposition were significantly increased after coating PES nanofibers with BG. In vivo studies demonstrated the formation of new bone at the defect site, and histological analysis results confirmed these observations [140,141].

In the studies, scientists have shown that electrospun PES nanofibrous scaffolds coated with BG are a promising substrate and can serve as osteoconductive implants for bone tissue regeneration [140,141].

**Figure 12:**
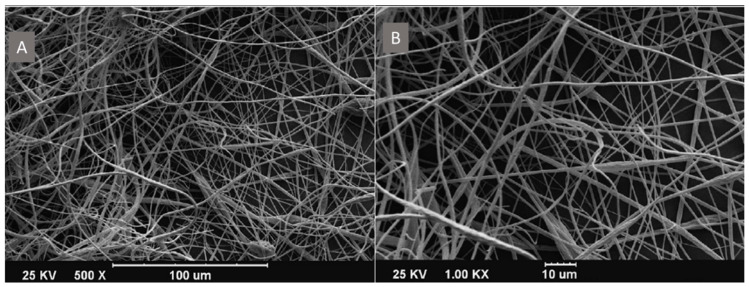
SEM imagine of PES/PANi nanofibers at two magnifications: (**A**)—x500 and (**B**)—x1000. Scale bar: (**A**) 100 µm; (**B**) 10 µm. The image was modified according to the literature [54].

As another example of PES-based nanofibrous materials in bone tissue engineering, a modified composite of PES with the addition of polyaniline (PES/PANi) was investigated, aiming at enhancing osteoinductivity (Figure 12). The PES membrane and composite were prepared via electrospinning. Subsequently, they were characterized in terms of morphology, mechanical, and physicochemical properties. The composite exhibited a significant difference in tensile strength compared to PES, while the mechanical stability of PES was markedly reduced after incorporation of PANi. Additionally, an increase in electrical conductivity was observed (PANi being a conducting polymer). The next step involved in vitro studies to evaluate the osteoinductive properties of scaffolds. Osteogenic differentiation of human mesenchymal stem cells (MSCs) was performed on the scaffolds. The highest growth, ALP activity, higher expression of type I collagen, osteonectin, and deposited calcium by cells were observed in PES-PANi groups compared to PES. It was demonstrated that the presence of PANi could stimulate cell activity leading to osteogenic differentiation. Higher osteogenic markers were detected in AT-MSCs cultured on PES/PANi compared to PES scaffolds. Thus, it was shown that the PES-PANi component exhibited promising potential as a bone graft substitute in tissue engineering [54,140].

Mahboudi et al. obtained PES nanofibers for the chondrogenic differentiation of bone-marrow-derived MSCs (BMSCs). The scaffold was fabricated via electrospinning. In vitro studies assessed the mRNA levels of cartilage-specific genes using RT-PCR after 21 days. The results demonstrated a significant increase in the expression of type II collagen and aggrecan genes on PES nanofibers. SEM imaging and immunocytochemistry were also conducted, confirming the differentiation of MSCs towards chondrocytes. The authors showed that the PES nanofibrous scaffold maintained the proliferation and differentiation of BMSCs into chondrocytes, which is applicable in cartilage tissue engineering [142].

In a separate investigation, undifferentiated human induced pluripotent stem cells (iPSCs) were grown on a PESscaffold composed of nanofibers, and their ability to undergo chondrogenesis was evaluated. Their findings indicate that the iPSCs cultured on the nanofiber scaffold exhibited enhanced chondrogenesis compared to the control group, demonstrating the highest potential for differentiation into cells resembling chondrocytes. Similar to the previous study, the authors demonstrated a higher expression of genes related to chondrogenesis, such as aggrecan, type II collagen, and type X collagen. This study shows that iPSCs and PES nanofibrous scaffolds are highly effective in treating cartilage damage and conditions [38,62].

In the next study, by Mansour et al., the use of modified PES nanofibers for assessing the differentiation of iPSCs towards pancreatic cells was presented. PES scaffolds were produced using electrospinning. To enhance hydrophilicity, they underwent surface plasma treatment under low-frequency (40 kHz) conditions, utilizing a cylindrical quartz reactor. This involved introducing pure oxygen into the reaction chamber at a pressure of 0.4 mbar, followed by applying glow discharge for a duration of 5 min. During an in vitro study, researchers demonstrated that the spatial PES matrix can provide a microenvironment that promotes the differentiation of iPSCs into insulin-producing cells (β cells). Higher expression of pancreas-specific markers at the mRNA and protein levels was observed compared to the tissue culture plate group. Furthermore, immunological testing revealed that the cells secreted C-peptide and insulin in response to glucose. The effectiveness of modified PES nanofibers in promoting the differentiation of iPSCs into β cells will be investigated in the perspective of in vivo studies [143].

**Figure 13:**
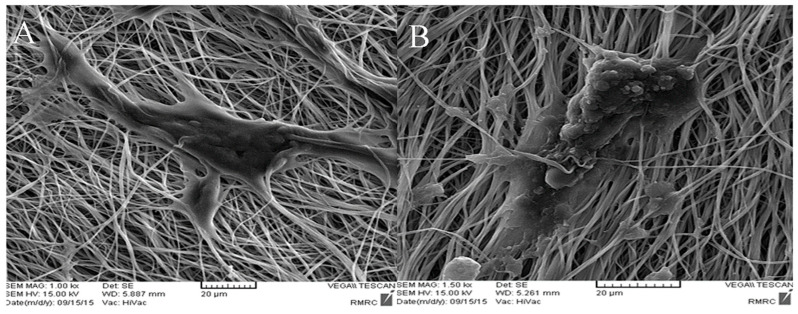
SEM imagine of iPSCs on PES/COL nanofibers: (**A**) before differentiation; (**B**) after 20 days of differentiation. The image was modified according to the literature [144].

In another study, Maymand and his research team investigated the hepatogenic differentiation of iPSCs on modified PES/COL nanofibers. The PES/COL nanofibers were obtained through the electrospinning method. Their surface was modified via plasma treatment (as described in the previous article [143]). Subsequently, they were coated with collagen via immersion in a solution of 1-ethyl-3-(3-dimethylaminopropyl) carbodiimide/N-hydroxysuccinimide for 12 h, followed by treatment with a 1 mg/mL solution of collagen I at 4 °C overnight. In vitro studies assessed the morphology and biochemical activity of iPSCs after 5 and 20 days of differentiation on aligned PES/COL nanofibrous scaffolds (Figure 13) compared to randomly selected nanofibrous membranes. RT-PCR and ICC analyses did not show significant differences in mRNA and protein levels of endodermal-specific markers. However, RT-PCR analysis revealed an increase in the expression of the *Cyp7A1* gene during differentiation on aligned PES/COL. An assessment was also made based on albumin production in conditioned media from hepatocyte-like cells differentiated on aligned PES/COL. The study demonstrated an increase in the expression of these markers after 20 days compared to randomly aligned nanofibers. Thus, this study indicates that aligned PES/COL nanofibrous scaffolds influence hepatogenic differentiation from iPSCs [144].

Currently, neural tissue engineering lacks an effective therapy for neuron regeneration. Ghollasi and his colleague designed modified nanofibrous scaffolds made of PES, upon which they conducted iPSCs culture. Random and aligned PES nanofibers were fabricated via the electrospinning technique. Subsequently, they underwent oxygen plasma treatment using a low-pressure device with oxygen discharge for 5 min (0.4 mbar, ~135 sccm, 40 kHz, 300 W). Prior to laminin immobilization, the samples were immersed in an EDC/NHS solution (5 mg/mL) for 12 h. Following this, the scaffolds were rinsed with deionized water and exposed to a laminin solution for 12 h at 4 °C. Then, in vitro studies assessed the ability of iPSCs to differentiate into neurons when cultured on random or aligned PES–plasma–laminin fibers. It was demonstrated that the modified fibers induced a significantly higher expression of neuronal genes compared to the untreated group. Moreover, they exhibited neurite elongation along the fiber direction and exponentially increased expression of neuron-specific markers such as early neuroectoderm marker enolase, axonal marker Tuj-1, and dendritic marker microtubule-associated protein 2. Simultaneous enhancement of hydrophilicity and biocompatibility, coupled with the utilization of topographical and directional cues, renders aligned PES-plasma-laminin scaffolds versatile for cell adhesion, proliferation, spreading, and iPSC differentiation into neurons. Designing scaffolds with desired physicochemical properties for cell adhesion, proliferation, and neuronal lineage differentiation may partially address current limitations. Therefore, the modified PES–plasma–laminin scaffold could serve as a versatile biomaterial platform with high differentiation potential for inducing neuronal differentiation of iPSCs and other cell lines in neurological disorders [145].

Below is a comprehensive table (Table 1) delineating the applications of PES material, which can be used in diverse forms. It has been demonstrated that its utility spans a wide spectrum of uses across both the medical and biotechnological domains.

## 4. Conclusions and Perspective

PES is a thermoplastic polymer. Its unique chemical and physical properties, such as excellent chemical resistance, high melting temperature, and mechanical strength, make it an attractive material for applications in various fields, from materials engineering to biotechnology and medicine. Its biocompatibility and ability to maintain structural integrity under physiological conditions make it an attractive material for medical applications. PES is used in the production of dialysis membranes, where its mechanical properties allow for the effective removal of toxins from the patient’s body. Additionally, PES has found application in the production of membranes for biological filtration and various types of bioreactors. Its excellent chemical resistance allows researchers to conduct biological processes in aggressive environments, which is crucial in the production of biomaterials and drugs. Furthermore, PES can be used as a membrane for biomolecule separation, which is applicable in various biotechnological processes, such as protein purification or the production of biological drugs.

Currently, the utilization of tools like clips in laparoscopic surgery derived from biomaterials has been steadily increasing, particularly in the field of urologic pathology. For kidney tumors in stages T1 and T2, laparoscopic nephrectomy is now advised as a procedure that yields results comparable to those of traditional methods. There are already approved and successfully used biomaterials, like PLC-Grena LTD Click’aV Plus ™ [146,147]. PES material can be an ideal candidate in this field. Another example of the future perspective for PES biomaterial is its potential use in developing a new type of biliary prosthesis aimed at preventing frequent post endoscopic retrograde cholangiopancreatography (ERCP) pancreatitis [148,149].

As can be observed, new applications of biopolymers in medicine and biotechnology are constantly emerging. Previous publications demonstrate the extensive use of PES, which could find even broader application in these fields due to its unique properties such as biocompatibility, mechanical strength, and chemical stability. Further research and efforts towards modifying this material may bring about innovative solutions that will positively impact the advancement of both science and medical practice.

In summary, PES has found application in medicine and biotechnology as a material with high biocompatibility and mechanical strength, enabling a wide range of implantation, diagnostic, and production applications in these fields. Its unique properties make it an attractive material for biomedical and biotechnological applications, contributing to the development of modern medical therapies and technologies.

## Figures and Tables

**Table 1:** The application of different forms of PES products in medicine and biotechnology.

PES Product	Application	Information
3D scaffolds	Cartilage regeneration—cell culture and laboratory study	In vivo study [43]
		In vitro study [30]
		Laboratory study [42]
	Liver tissue—cell culture	In vitro study [84]
		In vivo study [83]
HFMs	Kidneys—hemodialysis, blood purification application	In vitro study [92]
		Commercial membrane Purema [93,94]
		Commercial membrane ELISIO [95,96]
		PES-SS2 membrane—in vitro study [97]
	Liver tissue—cell culture	PES-carbon nanotubes composite HFMs—In vitro study with HepG2 [98]
		A three-dimensional liver tissue model—In vitro study with cryopreserved primary human hepatocytes [99]
	Endothelial cell culture	In vitro study with human endothelial cell growth [100]
	Pancreas—pancreatic islets in diabetic	In vitro and in vivo study—subcutaneous xenotransplantation of pancreatic islets in diabetic rats [104]
	Intestinal organoids—promise for the development of new drugs, nutraceuticals, food ingredients, and toxins	In vitro study with Coco-2 cells—model of PES HFMs and ECM components [109]
	Natural tissue—promise implant in neurodegenerative disease	A clinical study with Implant NsGene A/S [113] and the second-generation implant, NsG0202.1 [114]
Microcapsules	Drug delivery system—system for improving the feasibility of multi-drug delivery systems.	The system with two different docking units of microcapsules PES-g-PAAC and PES-g-PAAM [118]
	Drug release from aqueous core microcapsules	Alg/PES microcapsules—the process of protein diffusion from the alginate hydrogel core, through the polymeric wall [119,120]
Flat membrane	Stem cell culture	Fg-PES flat membrane as a versatile flat membrane for regenerative medicine [123]
	Device for encapsulation islet	Thin semipermeable flat membranes PES/PVP with a micropattern [124]
	Bioartificial kidney—“living membrane”	In vitro study with flat PES membrane coated by a double layer of L-DOPA and Coll IV [125]
	Tissue engineering and regenerative medicine	Biomimetic platform—modified PES membrane for the irreversible immobilization of bioactive proteins designed for cell culture [126]
	Blood purification, hemodialysis application	M3 flat membrane of PES and POC blending—in vitro study [127,128]
Nanofibers	Bone tissue engineering	The use of COL-PES nanofibers to heal and regenerate bone—in vitro study [139]
		PES nanofibers coated with bioactive glass—in vitro and in vivo study [140,141]
		Modified composite of PES/PANi—in vitro study [54,140]
	Cartilage tissue engineering	PES nanofibers for chondrogenic differentiation of bone-marrow-derived MSCs—in vitro study [142]
		iPSCs cultured on a PES nanofibers—in vitro study [38,62]
	Pancreatic islet—treatment of patients with type 1 diabetes mellitus	Differentiation of iPSCs towards pancreatic cells on modified PES nanofibers—in vitro study [143]
	Liver tissue engineering	The hepatogenic differentiation of iPSCs on modified PES/COL nanofibers—in vitro study [144]
	Natural tissue engineering	The modified PES–plasma–laminin nanofiber scaffold as a versatile biomaterial platform for inducing neuronal differentiation of iPSCs—in vitro study [145]
